# Two-Stage Method of Manufacturing Passive Conducting Structures by Laser Decomposition of AlN Ceramics

**DOI:** 10.3390/ma19194243

**Published:** 2026-10-07

**Authors:** Ryszard Pawlak, Marcin Lebioda, Leszek Klimek

**Affiliations:** 1 Institute of Electrical Engineering Systems, Lodz University of Technology, 90-924 Lodz, Poland; marcin.lebioda@p.lodz.pl; 2 Institute of Materials Science and Engineering, Lodz University of Technology, 90-924 Lodz, Poland; leszek.klimek@p.lodz.pl

**Keywords:** laser treatment, AlN ceramic, conductive Al paths, electronic structures, cryogenic temperature

## Abstract

This paper presents a two-stage method for fabricating passive conductive structures of arbitrary geometry on aluminum nitride (AlN) ceramic substrates via laser decomposition using nanosecond pulses of Nd:YAG fiber laser (*λ* = 1062 nm). We developed a *two-stage process* which allows one to create elements of low resistance and produce permanent joints to the obtained structures. Studies of laser decomposition of AlN under variable process conditions have shown that effective decomposition of the AlN ceramic surface layer should be carried out in an atmosphere of argon with laser beam fluence well above the ablation threshold. Notably, both processing stages were performed under a continuous flow of argon. A method of producing permanent joints to the obtained conductive structures was also developed. The electrical properties of the resulting conductive paths were evaluated over a temperature range of 20–300 K.

## 1. Introduction

Constructing electronic power devices while meeting the growing needs for miniaturization puts new demands on materials and chip packaging solutions. High-power electronic devices and hybrid electronic devices (modules) designed to work under harsh environmental conditions, such as automobiles, aircrafts and spaceships, have been intensively developed over the last twenty years. In such electronic devices, due to high values of dissipated power, aluminum oxide or aluminum nitride ceramics are used as substrates instead of traditional PCB (*Printed Circuit Board*) substrates [1,2,3,4,5,6]. The use of AlN ceramics is particularly advantageous due to their high hardness, high thermal and chemical stability, but above all due to their outstanding thermal properties: high thermal conductivity, even higher than 200 W/mK, and a thermal expansion coefficient close to that of silicon. In order to take full advantage of the insulating and thermal properties of ceramic substrates, it is necessary to metallize their surface, both for bonding with heat sinks or semiconductor structures and for contact-pad and conductive-path production [3,4,5,6]. The production of a copper, nickel or silver metallic layer on a ceramic or a polymer substrate in an electroless process or by sintering powder paste is often preceded by surface activation. The use of surface laser treatment has been found to be very effective for this purpose [7,8,9,10]. Recently, laser-assisted surface activation was performed on a 3D Al_2_O_3_ ceramic substrate that was produced by 3D printing [11,12]. In addition to the above-mentioned exceptional thermal properties, AlN ceramics have one unique feature: the laser treatment of the AlN surface causes the chemical decomposition of the material. By appropriate selection of process conditions, it is possible to produce an electrically conductive layer of pure Al directly on the insulating substrate. One of the first published reports on this subject was the work in [13]. Over the next 30 years, numerous studies on different aspects of the laser decomposition of AlN ceramics were carried out and published [14,15,16,17,18,19,20,21,22,23,24,25]. Numerous studies focused on the application of laser beam with different wavelengths, from UV (248 nm) [14,15,16], 266 nm, 355 nm [21,22,25], and visible radiation (532 nm) [21,25] to IR (1064 nm) [17,18,19,20,21,22,23,24,25] or 10.6 µm [16]. Various pulse durations from femtoseconds [16], picoseconds [22], nanoseconds [13,14,15,16,17,18,19,20,21,22,23] to microseconds [16] and pulse repetition frequency from several dozen Hz [15,21] to 1–200 kHz [13,16,17,18,19,20,21,22] were tested. Changes in other process parameters, such as the overlapping of pulses (60–95%), applied fluence and scanning speed of laser beams, were also tested. The laser-induced decomposition of AlN, which leads to the formation of a solid metallic layer of aluminum, is thermal in nature [16], so a more efficient way to create Al layers is the use of nanosecond pulses of Nd:YAG laser (wavelengths of 1064 nm) [13,17,18,19,20,21,22,23]. The formation of an Al layer as a result of the decomposition of AlN was used in a hybrid process of ceramic laser–chemical milling [26] and in the joining of a modified AlN surface with Cu [27]. However, the main goal of many studies was to use the laser decomposition of AlN to direct the patterning of conductive paths on a ceramic surface.

The suitability of laser decomposition of AlN ceramics for the direct production of created conductive paths has been evaluated by measurements of their electrical properties. Results have been presented in different ways: as values of electrical resistivity, conductivity, resistance of paths with specific dimensions, and surface resistance (resistance per square). The lowest electrical resistivity values obtained for Al paths (after approximate calculation of resistivity values based on published results of resistance and path dimensions) were: 1 × 10^−6^ Ωm [10], 0.25 × 10^−6^ Ωm [13], 0.5 × 10^−6^ Ωm [15], 1.72 × 10^−6^ Ωm [16], 0.64 × 10^−6^ Ωm [17,18], and 0.67 × 10^−6^ Ωm [21]. Laser decomposition processes of AlN which resulted in paths with low electrical resistivity were carried out in a protective atmosphere of Ar [13,17,18] or in vacuum [15,16,21]. Tests also confirmed the possibility of producing a conductive Al layer in a process carried out in air; however, the resistivity of such a layer was much higher (about 1 × 10^−5^ Ωm in [19]). Recent results of the laser decomposition process of AlN in air [22,23] also do not appear to be sufficiently satisfactory. With a very high pulse repetition frequency of 1000 kHz and substrate heating to a temperature of 350 °C [22], tracks with a thickness of 150 µm and a resistance of 0.26 Ω were obtained, giving an approximated resistivity of about 2 × 10^−6^ Ωm. The geometry of these paths, with grooves deeper than 100 µm, rather disqualifies their use as conductive elements in electronics. The paths obtained in [23] were also characterized by significant depression (60 µm) and deep grooves.

The resistivity values mentioned above indicate that 0.5 mm wide paths made in this way would have a resistance of less than 0.4 Ω per 1 mm of length; i.e., they would meet the condition for paths in high-power devices [16]. To the best of the authors’ knowledge, the practical utilization of laser-decomposed AlN to fabricate conductive structures remains unexplored in the existing literature. The cases described in the literature [10,13,14,15,16,17,18,19,20,21,22,23] were limited to straight paths of length ≤ 20 mm. Path resistance was measured using electrodes pressed against the path surface. There are no reports of permanent contacts to conductive paths made of Al. The few conductive structures produced by AlN decomposition of practical importance presented in the literature were not circuits and did not require the production of external leads [26,27]. Producing low-resistance and reliable joints is very important because circuit failure is usually caused by passive components or the packaging. The most important reasons causing difficulties in creating low-ohmic contacts to the obtained Al paths are the processes accompanying the decomposition of AlN. The Al surface becomes covered in an oxide layer very quickly, even when a protective gas is used. In order to obtain the lowest possible path resistance, the accumulated fluence of the absorbed beam should be large (more than 300–400 J/cm^2^) [17]. In such a process, on the path surface, a layer of redeposited debris is formed, the composition of which is complex and depends on the process conditions. The path material also contains contaminants.

The aim of the research presented in this article was to develop a technology for laser decomposition of AlN to enable the formation of permanent contacts to the fabricated conducting structures and the production of passive elements on the surface of AlN ceramics. This paper presents a method for the effective production of passive conductive structures of any geometry, made by nanosecond laser decomposition of AlN ceramics. We developed a *two-stage process* which is carried out in a protective atmosphere of argon. In the *first stage*, a conductive element is created under optimal conditions of AlN laser decomposition, allowing for low element resistance. In the *second stage*, debris is removed by laser ablation. During both stages, the ceramic sample is fixed, located in the chamber under a constant flow of argon, and is not in contact with the surrounding atmosphere. A method of producing permanent joints to the obtained conductive structures was developed. The electrical properties of the structures were tested in the temperature range 20–300 K.

To summarize, we developed a two-stage process for laser decomposition of AlN ceramic which allows one to create conductive paths and remove non-conducting debris and makes it possible to form permanent contacts to the paths.

## 2. Materials and Methods

**Figure 1:**
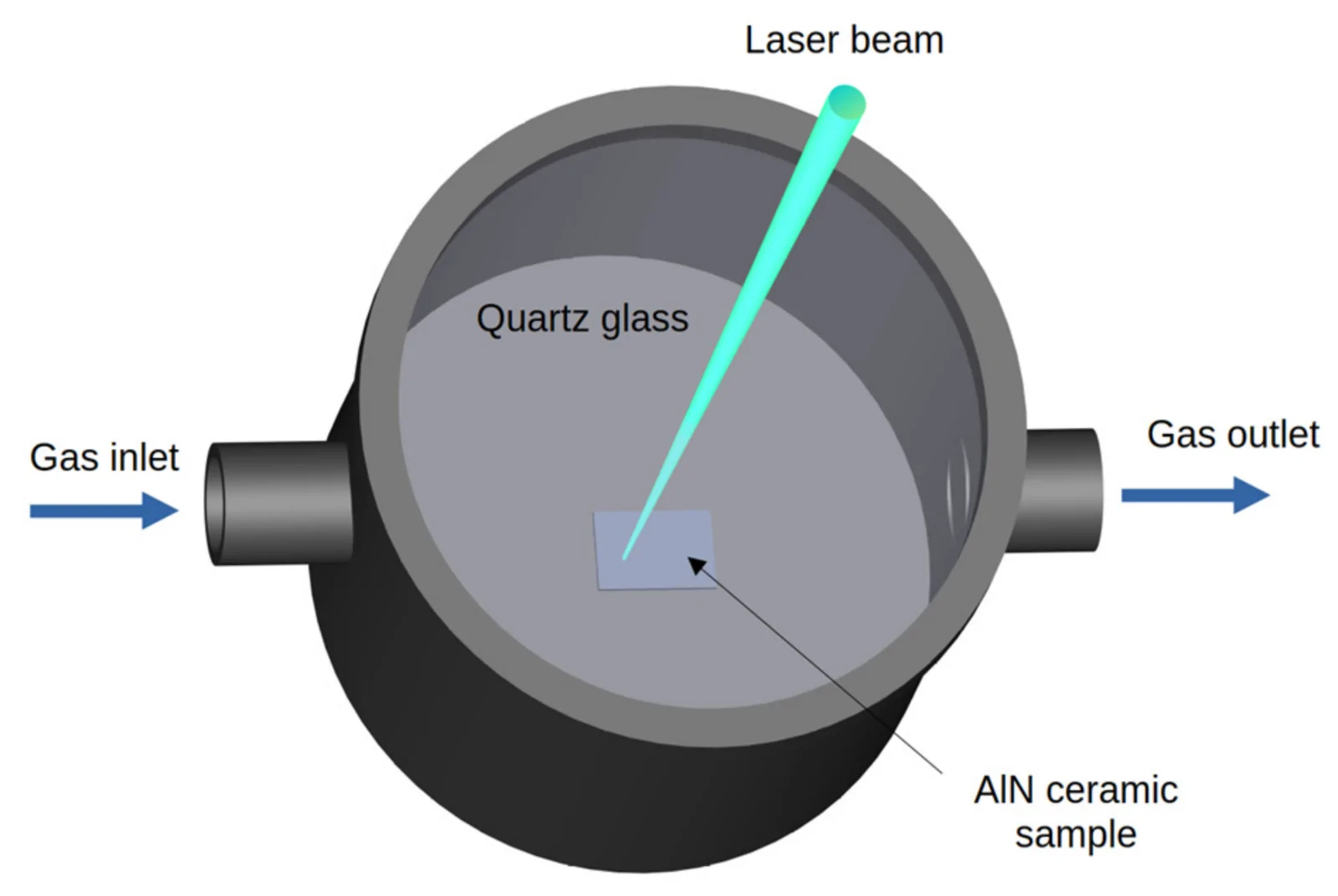
Schematic picture of reaction chamber for laser decomposition of AlN.

All tests were carried out using AlN plates with dimensions of 50 × 50 × 0.8 mm. The composition and structure of the substrates are presented in Section 3.1. Our studies used a laser micromachining system consisting of a Nd:YAG fiber laser (SPI G3 SM fiber laser, Southampton, UK) with an 8× expander, an F-theta lens (GEOMATEC Co., Ltd., Yokohama, Japan), focal length of 165 mm and a beam scanner (Xtreme beam scanner, Nutfield Techn. Inc., Londonderry, NH, USA). The optical system focused the laser beam to a 27 µm diameter area. The experiments were performed using laser pulses of durations of 220 ns, with a repetition frequency of 35 kHz. The laser system allows for adjustment of pulse energy from 0.09 mJ to 0.57 mJ and of beam scanning speed from 80 mm/s to 300 mm/s. While maintaining the same pulse repetition frequency, the overlapping *Ox* of laser pulses was increased by decreasing the beam scanning speed. Scanning speeds of 300 mm/s, 200 mm/s, 150 mm/s, 100 mm/s, and 80 mm/s correspond to an *Ox* equal to 68%, 79%, 84%, 89,4%, and 92%, respectively. Overlapping *Oy* equal to 63%, 68%, 74%, 79% and 84% corresponded to shifts in subsequent paths of 10 µm, 8.6 µm, 7 µm, 5.7 µm, and 4.3 µm, respectively. In the initial phase of this study, the process of laser decomposition of AlN was carried out in an open space of air or argon. The basic experiments were carried out in a chamber under a constant argon flow (Argon 5.0, Ar 99.999%, O_2_ ≤ 2 ppm, N_2_ ≤ 5 ppm, H_2_O ≤ 2 ppm,, CO_2_ ≤ 0.5 ppm, CH_4_ ≤ 0.5 ppm) of 22 L/min (Figure 1).

**Figure 2:**
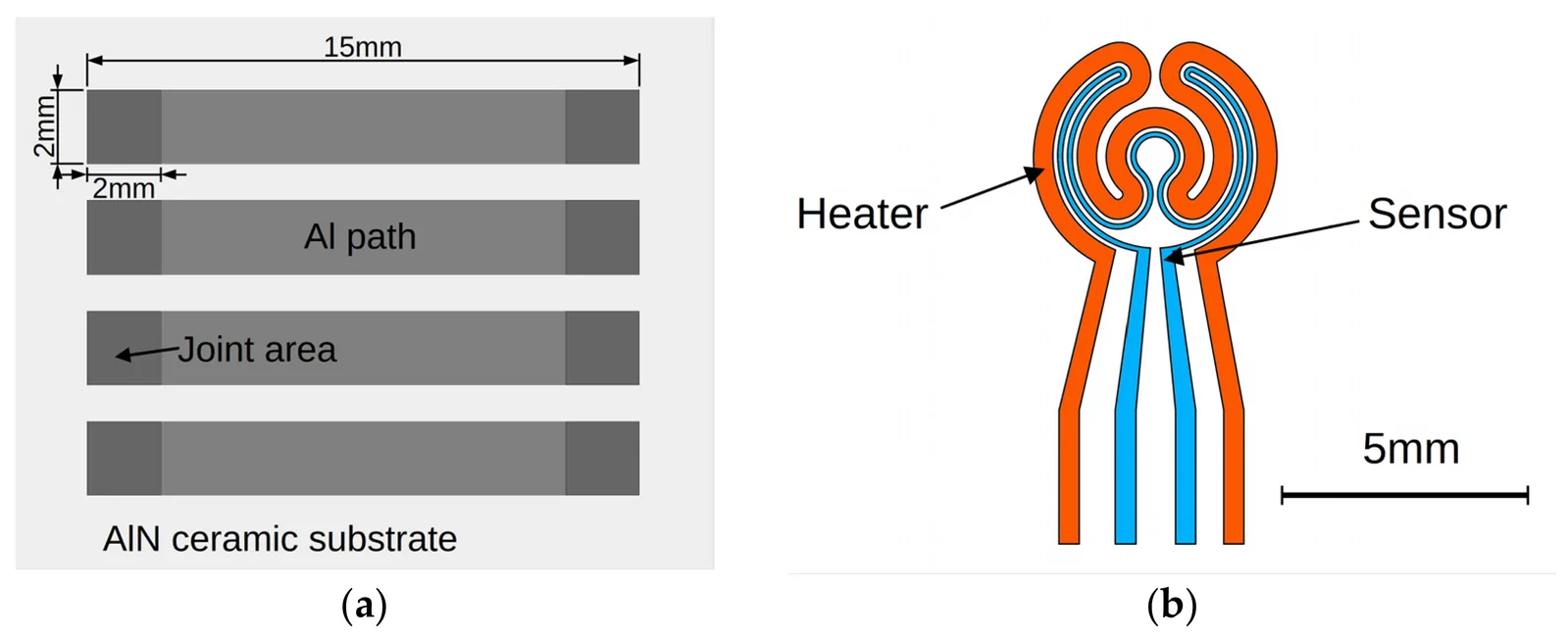
Sample geometries: (**a**) rectangular test paths; (**b**) heater structure with a temperature sensor.

The surface and structure of AlN ceramics and the layers formed by laser decomposition were examined using a VHX X1 confocal microscope (Keyence, Osaka, Japan) and a scanning electron microscope (JEOL JSM-6610LV (JEOL, Tokyo, Japan)) equipped with an EDS X- MAX 80 micro-analyzer (Oxford Instruments, Oxford, UK). The surfaces of the samples were subjected to SEM-BEIW observations and were imaged under an accelerating voltage of 20 kV. A PANalytical Empyrean X-ray diffractometer (Malvern Panalytical Netherlands, Almelo, The Netherlands) was also used for compositional analysis. Surface profiles were obtained by a Dektak XT- E profilometer (Bruker Corporation, Billerica, MA, USA). Most of the tests were performed on paths with simple geometry, in the form of a rectangle 15 mm long and 2 mm wide (Figure 2a). Silver tape electrodes (35 um Ag tape) were joined to the ends of the samples. Joints with an area of approximately 4 mm^2^ were made by using Elpox 15 (Amepox Microelectrocnis, Lodz, Poland) electrically conductive paste [28]. The voltage electrodes were point electrodes spaced 10 mm apart. This geometry facilitates the analysis of phenomena occurring during laser processing, as well as the performance of electrical measurements. Furthermore, using the same processing technology, a more complex heater structure with a temperature sensor was fabricated and tested at cryogenic temperatures (Figure 2b). This is an example of the practical application of the developed technology for the production of heating microstructures for cryogenic systems.

The measurement of the electrical properties of Al paths on AlN ceramic substrates were performed at room and cryogenic temperature. The four-probe method was used to measure sample resistance using a Keysight 34420A microohmmeter (Keysight Technologies, Santa Rosa, CA, USA). The measurements at cryogenic temperature were performed in a helium closed-cycle DE-210 cryostat (Advanced Research Systems Inc., Macungie, PA, USA) [29,30]. Samples were cyclically cooled in a vacuum chamber from 300 K to 20 K at the rate about 4 K/min. The samples were placed on a massive copper heat exchanger so that the entire surface of the samples had good thermal contact with them. This arrangement eliminated temperature gradients within the samples. The heat exchanger was fixed directly on the “cold finger” of the cryocooler. The temperature of the samples was controlled and measured with the reference temperature sensor, a DT-670-SD silicon diode (Lakeshore Cryotronics Inc., Westerville, OH, USA), and with a Lake Shore 331 temperature controller (Lakeshore Cryotronics Inc., Westerville, OH, USA).

## 3. Results and Discussion

Many papers [13,16,17,19,21,23,24] have analyzed the dependence of the morphology, dimensions, and electrical properties of the Al layer on the parameters of the laser decomposition process of AlN. Attempts have even been made to establish functional relationships between the process parameters and the electrical resistance of Al layers [10,23]. Although the research results of various authors are convergent, their comparison does not allow for quantitative generalization. In our opinion there are many factors contributing to this state of affairs. The phase composition of a given ceramic species, which determines the absorption of the first laser pulses, as well as varying pulse durations and repetition rates (different accumulation of absorbed energy), consequently influences the kinetics of thermal processes. The results of the AlN decomposition process will therefore differ quantitatively due to the different course of reactions occurring in the laser beam interaction region. The main goal of the undertaken research study was to obtain aluminum paths with a low surface resistance value (*R_sq_*) (high electrical conductivity) and appropriate surface morphology suitable for joining solid leads. Therefore, below we present the results of our research focused on determining the influence of selected factors with varying degrees of variability, ensuring the achievement of the assumed goal.

The laser decomposition process, carried out both in air and with free Ar flow, resulted in the formation of an Al layer on the ceramic surface. The obtained layers, depending on the accumulated laser beam fluence from 50 to 800 J/cm^2^, were covered with a deposit ranging in color from light brown to dark brown. Their surface resistance, measured using point-contact electrodes, was in the order of 0.1–0.6 Ω/sq. The mechanical removal of the deposit layer led to the destruction of the Al layer, preventing the use of any technology for creating leads. Further research was therefore conducted using a chamber with a continuous argon flow, employing the *two-stage process* described in Section 3.2 and Section 3.3.

### 3.1. Structure and Composition of AlN Ceramic Substrate

**Figure 3:**
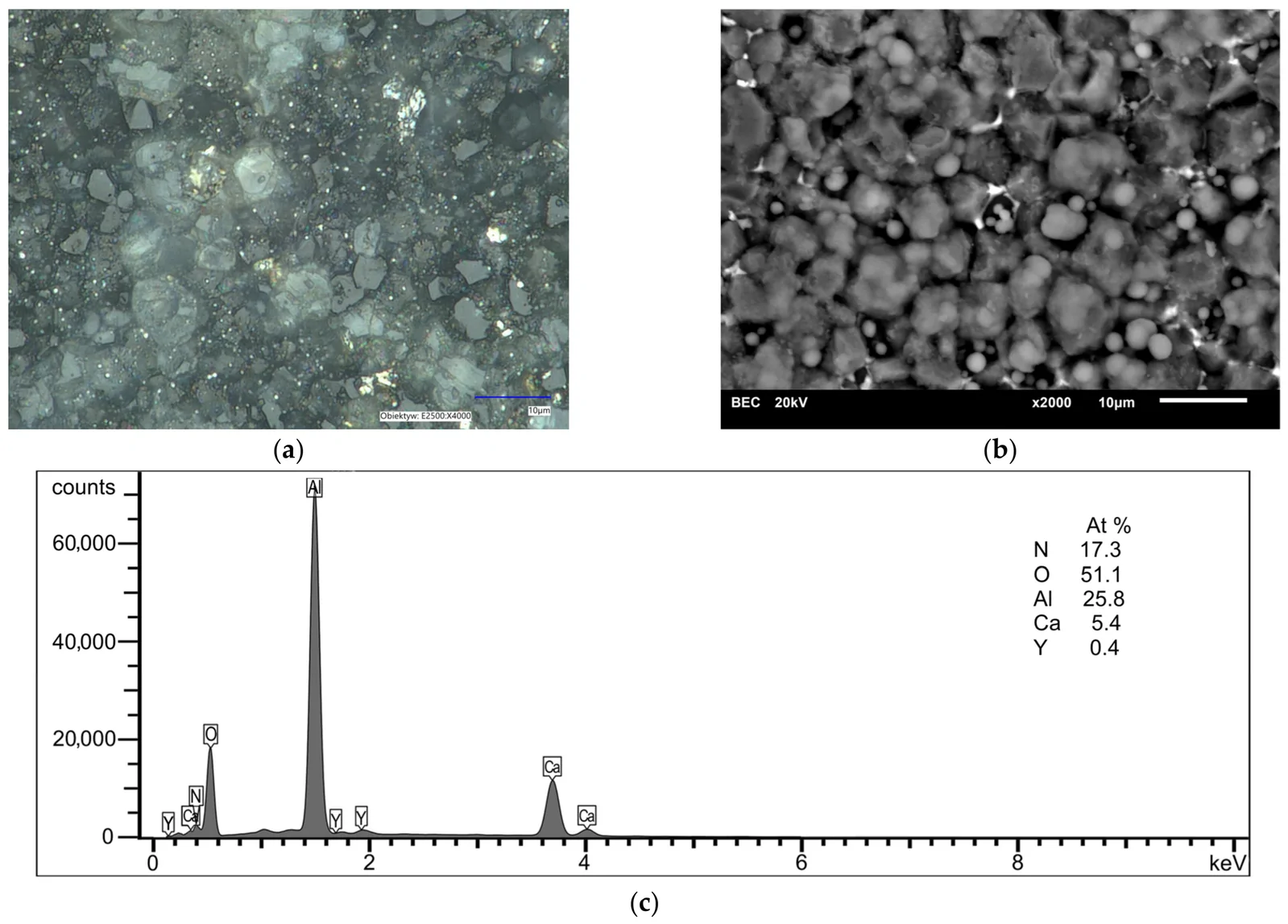
Surface of AlN ceramic substrate: (**a**) digital microscope image of surface of AlN plate; (**b**) SEM (*scanning electron microscope*) picture of surface of AlN plate; (**c**) EDS (*energy-dispersive X-ray spectroscopy*) spectrum of AlN ceramic substrate surface.

**Figure 4:**
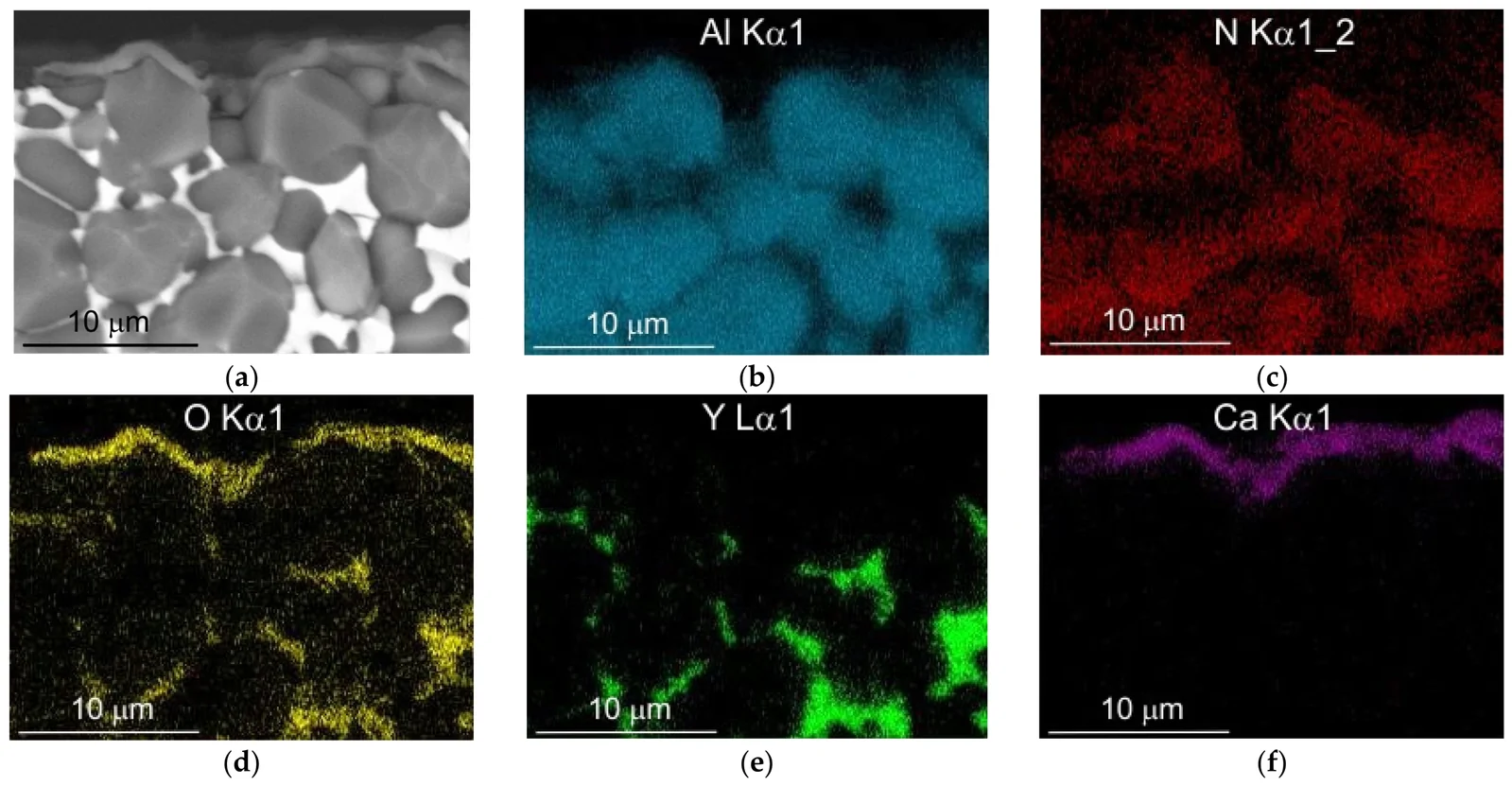

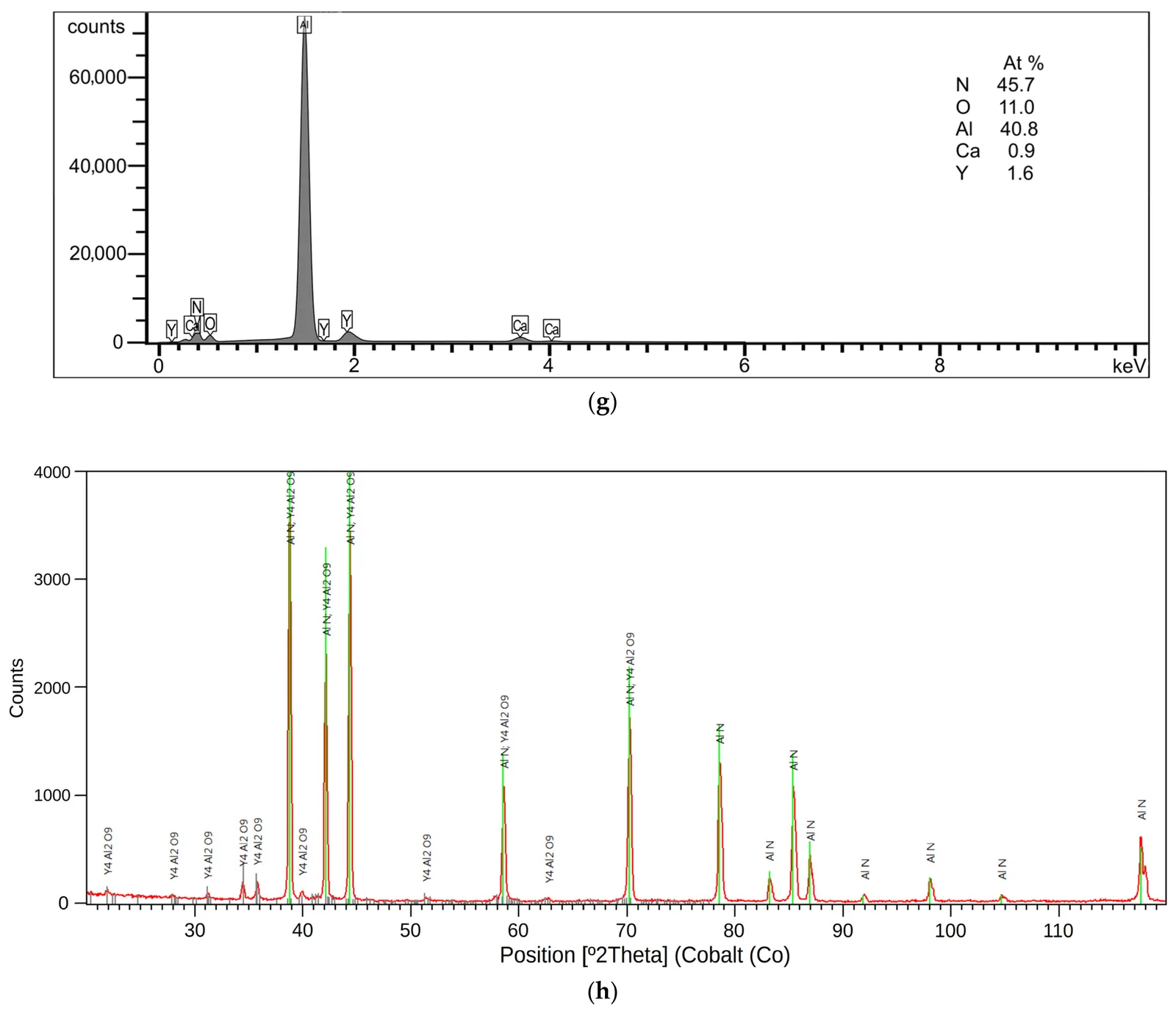
Composition of AlN ceramic substrate: (**a**) SEM picture of transverse section of AlN plate; (**b**–**f**) content map of Al, N, O, Y, and Ca, respectively; (**g**) EDS spectrum of AlN ceramic substrate section; (**h**) diffraction pattern of AlN ceramic.

Detailed studies of the AlN ceramic used for this research study were performed; they were necessary for the analysis of the course of each stage of the process and the final effects of AlN decomposition. Microscopic images and composition of an AlN ceramic substrate plate are shown in Figure 3 and Figure 4. The pictures of the ceramic substrate surface obtained with both an optical and a scanning electron microscope (Figure 3) and the SEM picture of the substrate cross-section (Figure 4a) show the fine-grained structure of the AlN ceramic used in this study. The diameter of the ceramic grains is approximately less than 5 µm. The elemental content maps (Figure 4b–f) indicate the presence of Al, N, O, Y, and Ca in the ceramic substrate. This is confirmed by the results of the EDS analysis performed on both the surface (Figure 3c) and the cross-section (Figure 4g) of the ceramic sample. The ceramic substrate used for the tests consists mainly of aluminum and nitrogen. During the production of AlN ceramics, yttrium and calcium are added, among others, because they form stable oxides segregating at the grain boundaries [31,32]. This reduces the oxygen content in the AlN phase and increases the thermal conductivity of ceramic and its mechanical strength. Interestingly, yttrium oxides are distributed at the AlN grain boundaries (Figure 4d,e), while calcium oxides are distributed on the surface of the ceramic plate (Figure 4d,f).

The concentration of calcium oxides in the surface layer approximately 1 µm in thickness (Figure 4a,f) explains the compositional differences between the results of the EDS analysis of the surface (5.4 at% Ca and 0.4 at% Y; Figure 3c) and of the sample cross-section (0.9 at% Ca and 1.6 at% Y; Figure 4g).

### 3.2. Effects of Laser Decomposition of AlN Ceramic After the First Stage of the Process

The intensity of the laser decomposition process of AlN ceramics depends on the fluence value (surface energy density). For a single laser pulse, it is defined as:

(1) $$F_{p}=\frac{E_{p}}{\pi d_{0}^{2}}$$

where *F_p_*—single-pulse fluence; *E_p_*—laser pulse energy; and *d*_0_—diameter of focused laser beam.

In the process of generating a path using a laser beam with a pulse repetition frequency *f_rep_* scanned linearly at a velocity *v_S_*, the phenomenon of energy accumulation of individual pulses, partially overlapping on the material surface, plays a significant role. Therefore, the average accumulated fluence value is determined, which is defined as:

(2) $$F_{\mathrm{AV}}=\frac{f_{\mathrm{rep}\;}E_{p}}{v_{S}d_{0}}$$

The overlapping *Ox* in the beam scanning direction is defined by the relationship:

(3) $$O_{x}=\left(1-\frac{v_{S}}{f_{\mathrm{rep}\;}d_{0}}\right)\times 100\%$$

If the path is produced by the partial overlapping of successive scans of the laser beam, the overlapping *Oy* is defined as:

(4) $$O_{y}=\left(\frac{d_{0}-h}{d_{0}}\right)\times 100\%$$

where *h*—the shifting of successive scans of the laser beam.

**Figure 5:**
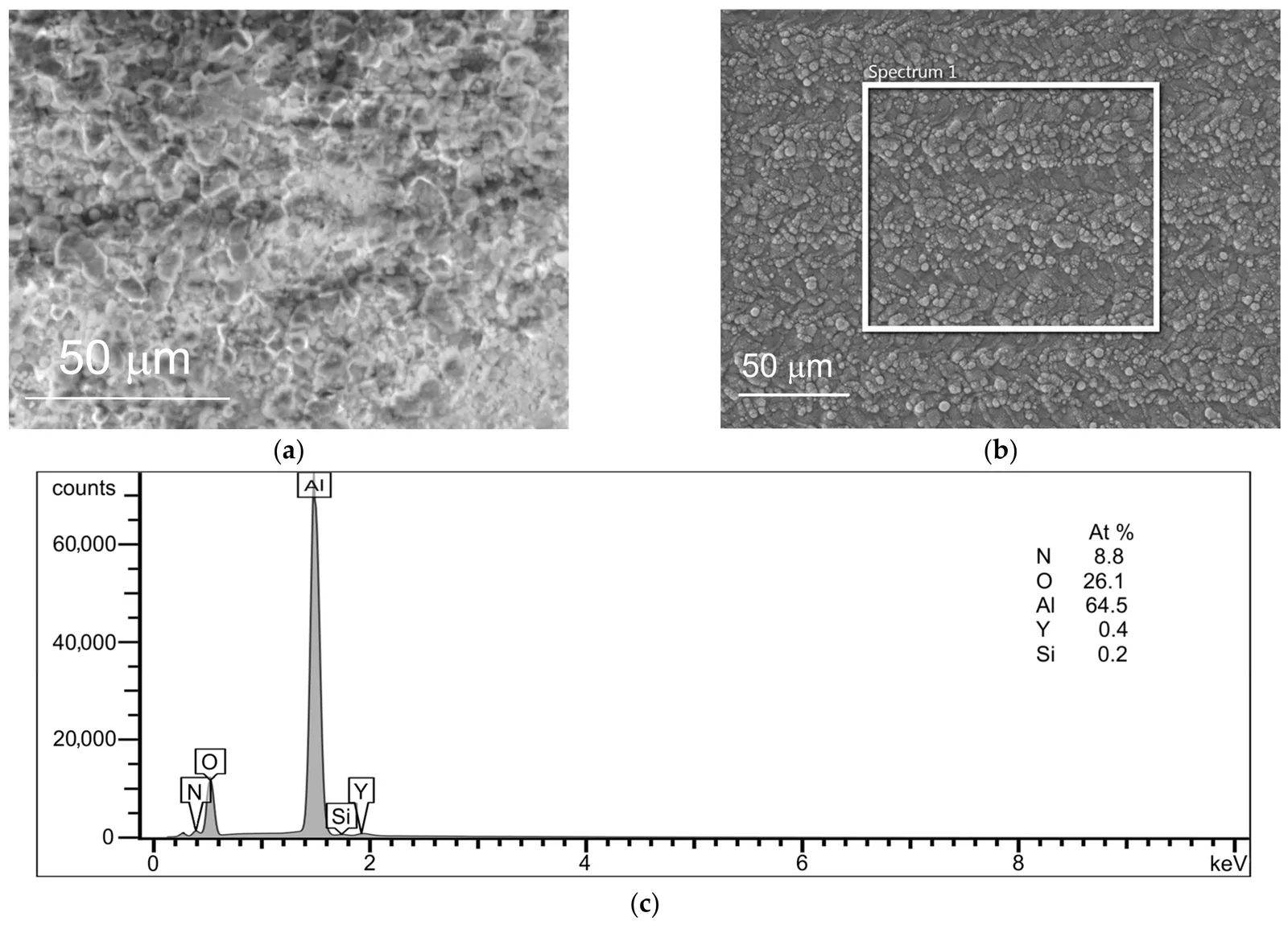

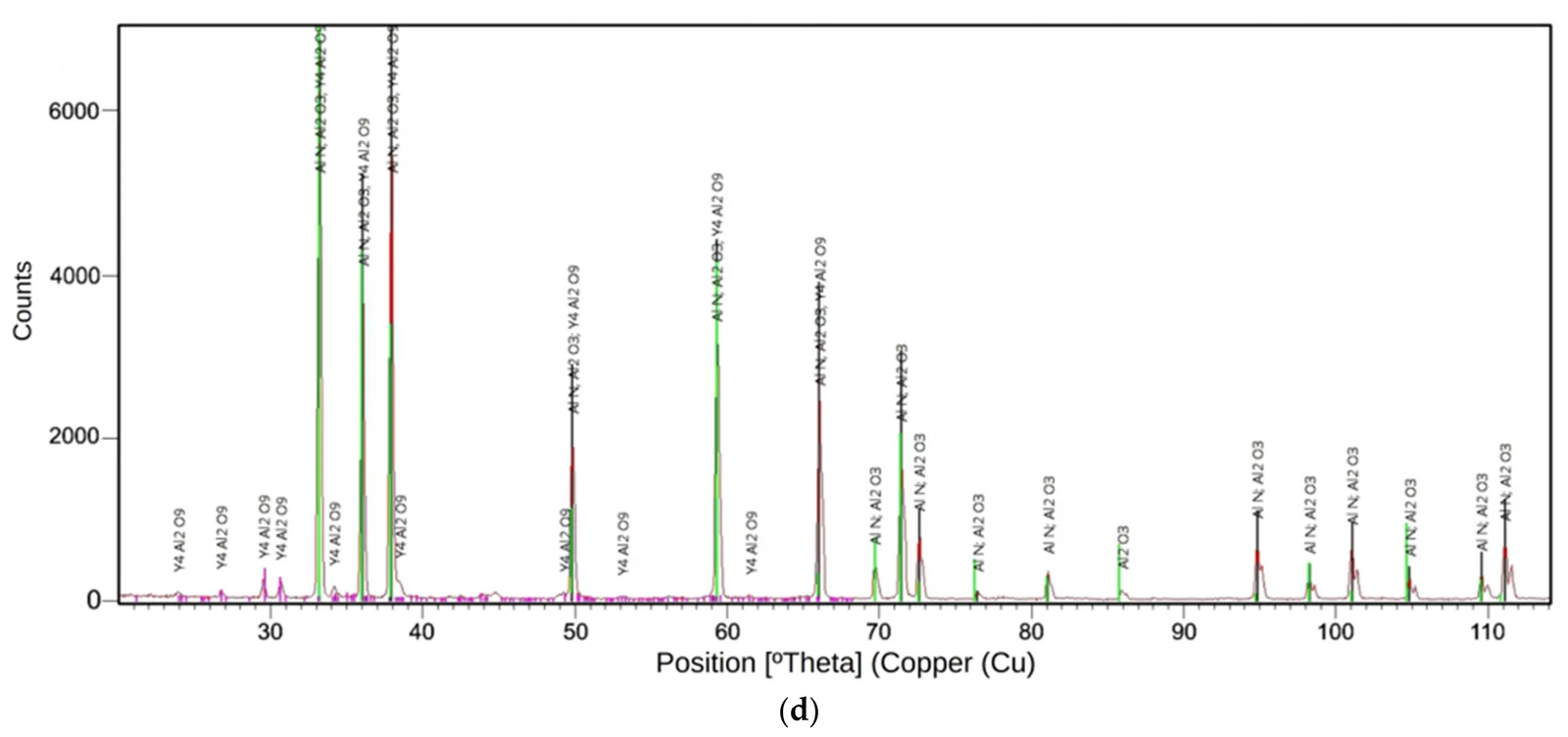
Ceramic substrate before and after laser treatment: (**a**) SEM image of ceramic substrate surface not laser-processed (spectrum in Figure 3c); (**b**) SEM image of black deposit on the surface after *first stage* of the process; (**c**) EDS spectrum of the black deposit; (**d**) diffraction pattern of the black deposit layer.

During the first stage of the process, carried out in a chamber under continuous Ar flow, a decomposition process occurred on the surface of AlN ceramic when the value of accumulated laser beam fluence (*F_AV_*—*average accumulated fluence*) reached a level of approximately 75 J/cm^2^. As a result of the aluminum nitride decomposition reaction, a black, porous layer covered the surface of the generated Al tracks (Figure 5b). EDS analysis revealed the presence of aluminum, oxygen, nitrogen, and trace amounts of yttrium and silicon in the black deposit (Figure 5c). This black layer is non-conductive because it contains aluminum in the form of Al_2_O_3_, AlN, and Y_4_Al_2_O_9_ compounds (Figure 5d).

The black top layer posed a problem in the practical application of the obtained Al paths. Its mechanical removal proved to be only partially effective, due to the roughness of the aluminum layer, among other things. Electrical conductivity measurement of the Al paths after *the first stage* of the process showed that only strongly pressed tip electrodes provided good contact with the aluminum layer underneath the black deposit and ensured stable measurement. The sheet resistance *R_sq_* values were very small, at the level of 0.08 Ω/sq. Effective removal of the black deposit was ensured by the repeated scanning of the traces with a laser beam (*the second stage*). It should be emphasized that in the *first and second stages* of the process, the substrate was in a chamber under continuous argon flow, without any contact with air.

### 3.3. Effects of AlN Ceramic Decomposition After the Second Stage of the Process

The re-scanning of the path with a laser beam in *the second stage* of the process resulted in the ablative removal of the black deposit, revealing the metallic, shiny aluminum surface underneath. Due to the high absorption (resulting from the color and morphology of the black deposit), the accumulated laser beam fluence required for effective ablation was more than two times lower than the threshold value, causing the decomposition of the ceramic in *the first stage* of the process.

Further investigations utilized a laser beam with the following constant parameters: laser pulse duration *t_p_* = 220 ns and pulse repetition frequency *f_rep_* = 35 kHz. The pulse energy of the laser has a Gaussian distribution, so the focused beam diameter *d_0_* = 27 µm was defined at the level of 1/e^2^. The variable parameters in the *first* and *second stages* of the process are presented in Table 1.

The accumulated fluence (Table 1) calculated based on relationship (2) refer to the creation of one path in the *x* direction. The lowest pulse energy value for the process (pulse overlap *Ox* = 68%; v_S_ = 300 mm/s), at which the decomposition of AlN ceramics takes place and an electrically conductive path is created, is 0.170 mJ, which corresponds to an accumulated fluence of 74 J/cm^2^. The accumulated fluence value in the second stage of the process was significantly lower and amounted to only 43 J/cm^2^. Optimal conditions for the *second stage* (Table 1) were experimentally determined. Single-pulse fluence for the lowest and highest pulse energy values used in the tests (Table 1), calculated based on relationship (1), were 29.5 J/cm^2^ and 99.5 J/cm^2^, while for the second stage of process, the value was only 17.5 J/cm^2^.

#### 3.3.1. Surface Topography and Thickness of Al Layer

**Figure 6:**
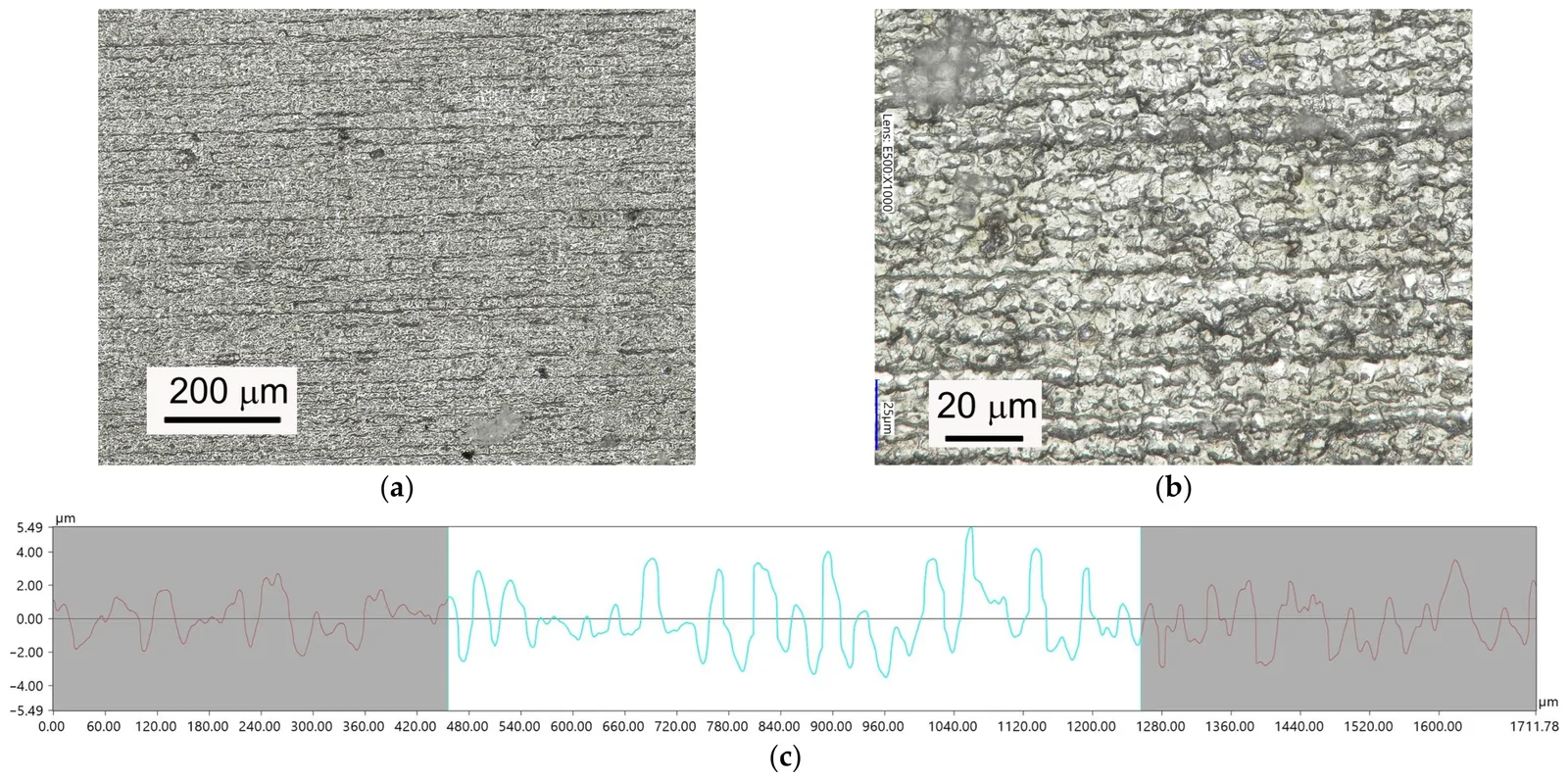
Surface topography of Al layer (carried out with *E_p_* = 0.456 mJ, *v_S_* = 0.3 m/s, *F_AV_* = 197 J/cm^2^, *O_x_* = 68%, and *O_y_* = 68%): (**a**,**b**) digital microscope image at magnification of 100× and 1000×, respectively; (**c**) sample roughness profile perpendicular to the path, with *R_a_* = 1.50 µm.

The surface geometry of the aluminum paths reflects the process sequence of the laser decomposition of AlN ceramic (Figure 6a,b). The effects of the interaction of individual laser beam trajectories, repeated with an appropriate overlap coefficient (*Oy*), are particularly visible.

**Figure 7:**
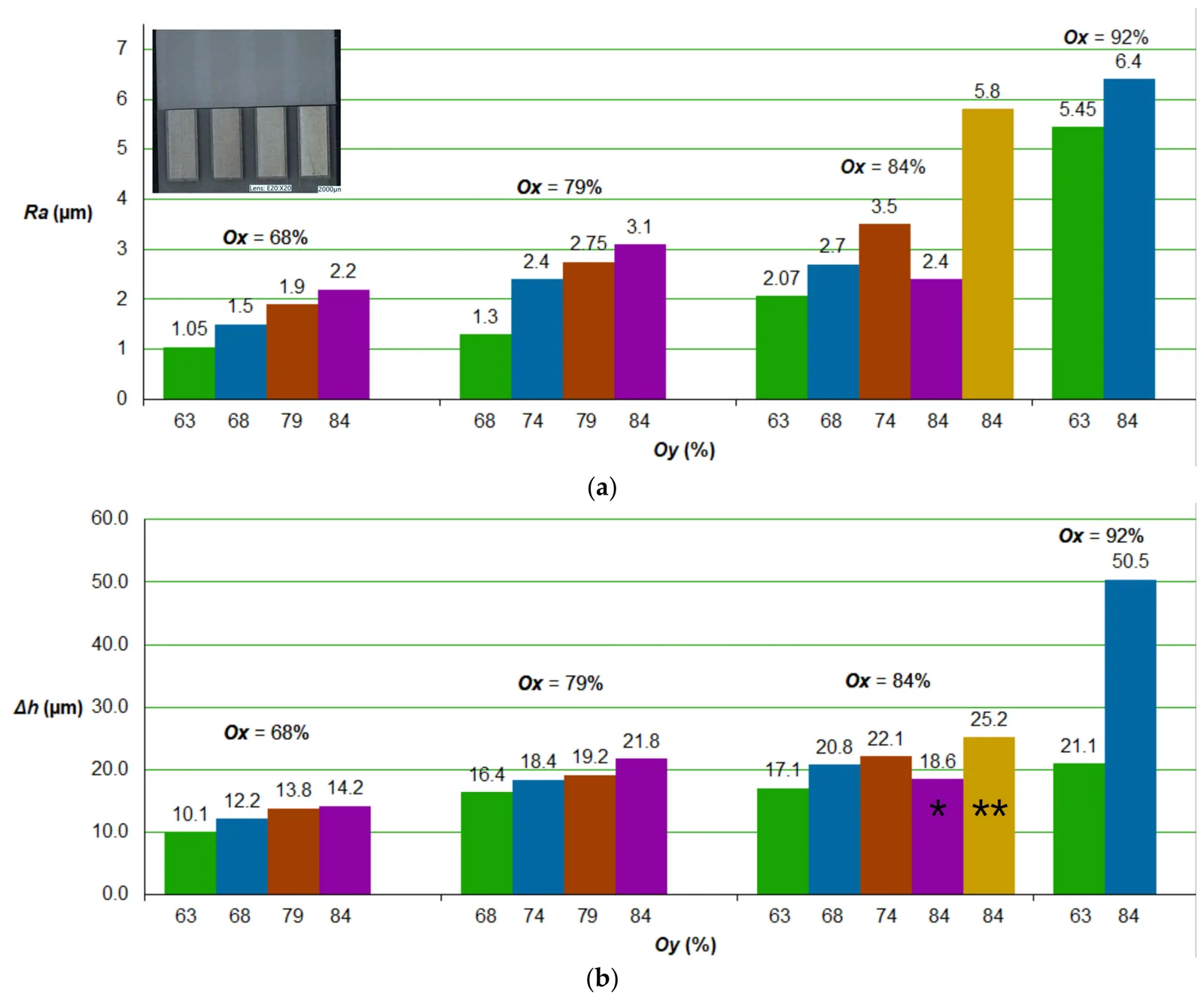
Surface geometry of Al paths: (**a**) smoothness of the Al layer surface—roughness parameter *R_a_* along line ⊥ to the Al path for selected parameters of the decomposition process (inset: digital microscope image of substrate with Al paths (below) and after the chemical etching of Al paths (above)); (**b**) the depression of the paths for different conditions of *the first stage* of the process. Symbols (*) and (**) correspond to transverse and longitudinal laser scanning directions relative to the track length, respectively.

The surface smoothness and transverse dimensions of the produced Al paths were determined based on the roughness profile of samples containing Al layers (Figure 7). For this purpose, the profiles of the Al path and the bottom of the cavity after the chemical etching of the path were compared.

The path surface roughness increases with the increase in the value of accumulated laser beam fluence *F_AV_*, which is caused both by the increase in laser pulse energy and by the increase in the degree of overlap (*Ox* or *Oy*). This is confirmed by the values of the roughness parameter *Ra* measured perpendicularly to the path (Figure 7a). From the perspective of the functional application of the obtained conductive paths as elements of electronic circuits, the depression of their surface relative to the initial surface of the AlN ceramic is significant. The minimum depression of the conductive aluminum path that can be achieved will have a decisive influence on the possibility of creating electrical connections to the path. The variations in the depression of the paths for selected conditions of *the first stage* of the process are shown in (Figure 7b). Based on Figure 7b, it can be concluded that for a moderate degree of overlapping *Ox* = 79% and *Ox* = 84%, which correspond to the accumulated fluence values of 296 J/cm^2^ and 394 J/cm^2^, the depression increases with the increase in *Oy*. For *Ox* = *Oy* = 79% and *Ox* = *Oy* = 84%, it is in the range of approximately 15 µm to 25 µm. This path depression is smaller than, for example, that in [17]—approximately 80 µm for 400 J/cm^2^. It should be noted that in the studies in [17], a train of pulses with a duration of 45 ns repeated at a frequency of 80 kHz were used, which enhanced the ablative effect of the beam. For the case of *Ox* = *Oy* = 84%, two bars, (*) and (**), are provided for comparison in Figure 7b. The average Al path depression of 18.6 µm (*) refers to the process in which the laser beam was scanned transversely to the sample length, while (**) was created by scanning along the path.

**Figure 8:**
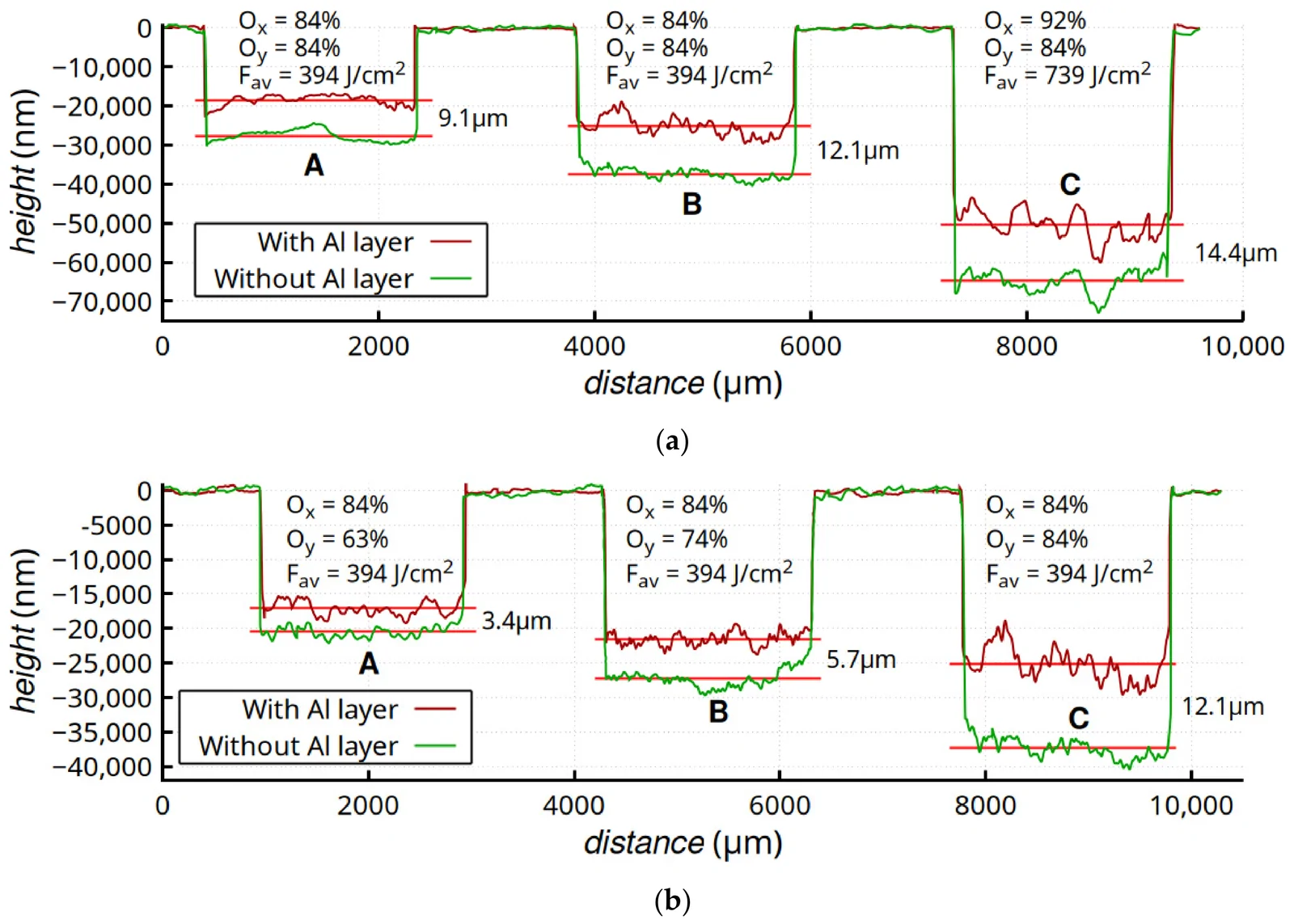
Comparison of the surface profiles of the Al paths and the cavities after chemical etching: (**a**) profiles for different *Oy* and *F_AV_* values; (**b**) profiles for constant *F_AV_* value.

Although, in this case, the Al path surface (*) is less recessed, it has smaller thickness and higher resistance (compare Figure 8a, profiles A and B). It is worth emphasizing the high convergence of changes in the values of *Ra* (Figure 7a) and Δ*h* (Figure 7b) for the same variants of the parameters *Ox*, *Oy* and *F_AV_*. Figure 8a,b summarize the profiles of the surface and the bottom of the layer for selected parameters of *the first stage* of the decomposition process. The images provide averaged profiles for the specific process conditions which allowed for the determination of the geometric mean thickness of the Al layer. The thickness values given in the figures were calculated for three measurements in each of five samples. Precise determination of Al layer thickness based on measured profile parameters is difficult due to significant peaks and valleys, both on the path surface and at the bottom of the layer (the trace on the ceramic substrate after chemical etching). It is clear that using the described measurement procedure, only an approximate determination of the Al track thickness is possible. The analysis of selected examples presented in Figure 8a,b shows that increasing the overlap of pulses *Ox* causes an increase in layer thickness (profiles B and C in Figure 8a), with extremely large *Ox* being associated with large unevenness of the Al layer. Scanning the ceramic surface along the assumed path geometry allows one to obtain a layer of greater thickness (Figure 8a, profile B) than in the case of transverse scanning (Figure 8a, profile A). For the same *Ox* value, greater overlap *Oy* of subsequent laser beam trajectories increases the layer thickness (profiles A, B, and C, Figure 8b), with a simultaneous increase in surface unevenness. In the AlN decomposition processes for different parameter variants presented in Table 1, layers with thicknesses of 0.8 to 1.2 µm (*Ox* = 68%, *Oy* = 68%, and *F_AV_* = 74 J/cm^2^) to 16 µm (*Ox* = 92%, *Oy* = 84%, and *F_AV_* = 924 J/cm^2^) were obtained.

#### 3.3.2. Composition and Structure of Al Layer

**Figure 9:**
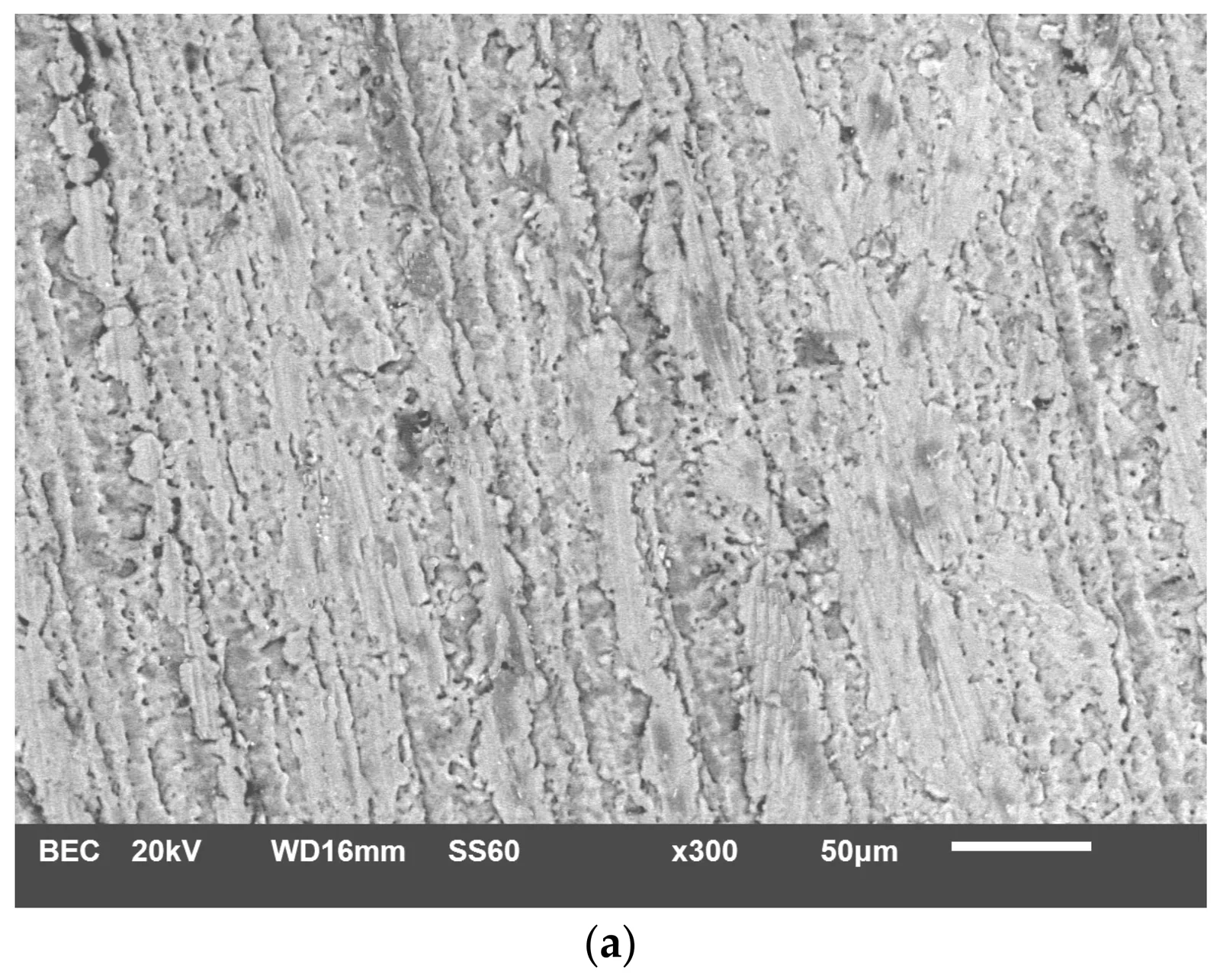

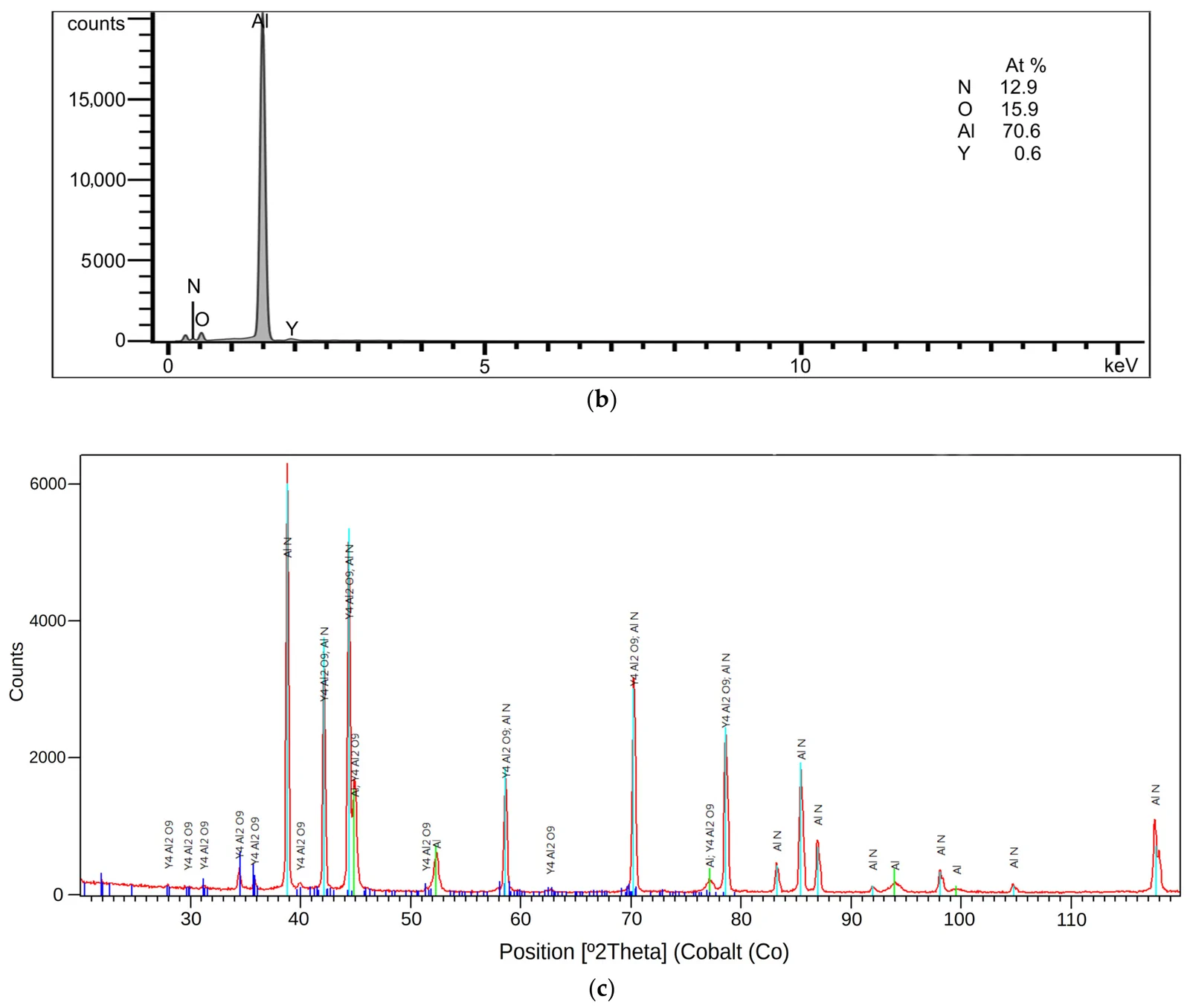
Al path after the *second stage* of the process (carried out with *E_p_* = 0.456 mJ, *v_S_* = 0.2 m/s, *F_AV_* = 296 J/cm^2^, *O_x_* = 79%, and *O_y_* = 79%): (**a**) SEM picture of aluminum path surface; (**b**) EDS analysis of Al path composition; (**c**) diffraction pattern of aluminum path.

The highly developed surface of the Al path shown in Figure 9a resulted from the nature of the laser decomposition process, which, in the case of nanosecond laser pulses, is thermal ablation. Various reactions can occur during this process, but one of them results in the formation of a liquid aluminum phase, which, together with condensing Al vapor, forms a conductive layer [16,18]. Other reactions that occur include the presence of AlN and N in the gas phase. Their presence and the enormous heating kinetics of 10^9^ K/s determine the turbulent course of phenomena in the reaction zone, contributing to significant surface irregularities.

EDS analysis of the Al layer (Figure 9b) showed a very high aluminum content (70.6%), significantly higher compared with the previously published results of laser decomposition of AlN ([10]—57%; [16]—60%; [17]—42%; [18]—41%). Compared with the composition of AlN ceramics, the N content was reduced from 45.7% to 12.9%. This is indicative of the metallic nature of the path material with non-conductive inclusions, e.g., Al_2_O_3_, AlN, and Y_4_Al_2_O_9_. The X-ray diffraction (XRD) pattern (Figure 9c) confirms the presence of metallic Al. The low intensity of the Al diffraction peaks is attributed to the X-ray penetration depth, which encompasses both the Al top layer (5–15 µm in thickness) and the underlying ceramic substrate. Consequently, characteristic reflection peaks from the substrate material are prominently visible on the diffractogram (Figure 4h). Due to the low ratio of the Al layer thickness to the probed volume of the ceramic substrate, the resulting Al reflections remain weak. In contrast, energy-dispersive X-ray spectroscopy (EDS) analysis (Figure 9b) provides a more reliable quantitative assessment of the Al content within the synthesized layers. This is because the EDS interaction volume (signal depth) is confined to a few micrometers—a depth comparable to or smaller than the layer thickness itself.

### 3.4. Electrical Properties

**Figure 10:**
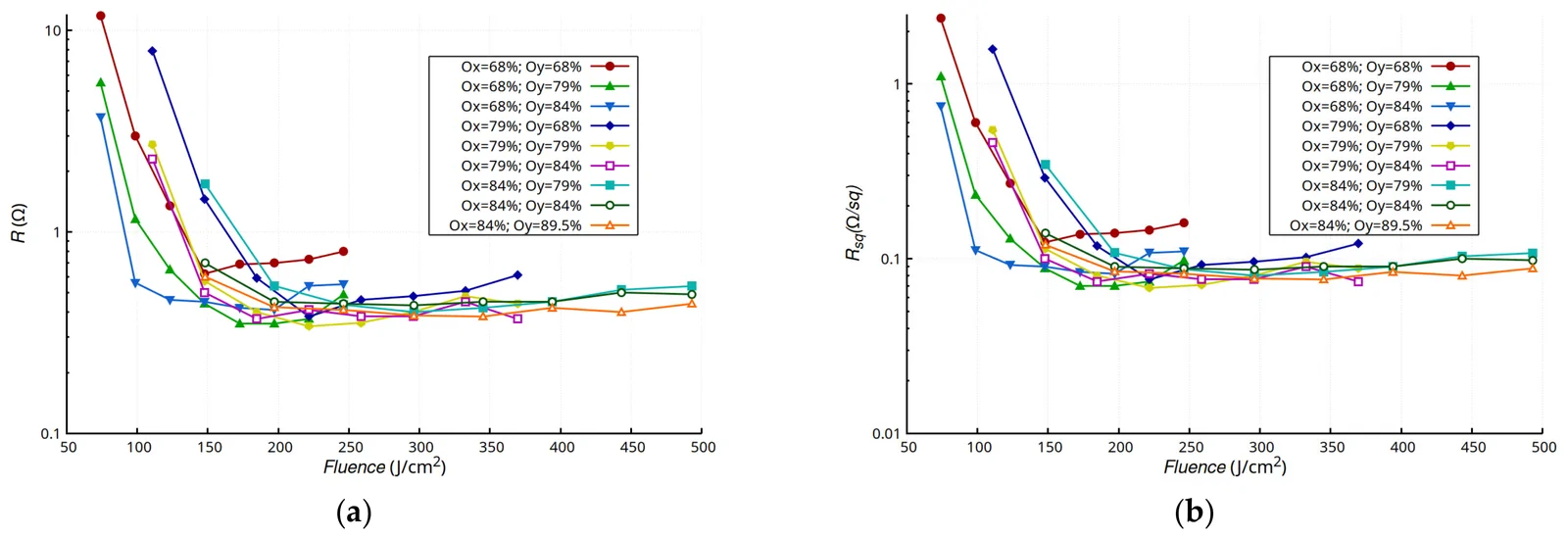
Effect of laser beam fluence and of overlap of laser pulses (**a**) on the resistance of 12 × 2 mm samples and (**b**) on surface resistance.

The results of the dependence of resistance on fluence (Figure 10) show that the lowest resistance was obtained at a fluence of 250 J/cm^2^ and higher. These results are directly correlated with the estimated Al layer thickness (Figure 8) and the lowest layer roughness coefficient *Ra* (Figure 7). The layers obtained for these laser processing parameters are characterized by relatively large thickness and lower roughness, which result in a lower electrical resistance value. Based on the presented results, it could be concluded that the above parameters can be accepted as indicators of the quality of the obtained conductive paths. It should be noted that a significant increase in fluence, which caused, among others, an increase in depression (Figure 7b and Figure 8a), does not significantly affect the electrical parameters of the layer (Figure 10). The morphological parameters of the layers changed, but no destruction of the layer or loss of electrical conductivity was observed. The aim of our research was to produce conductive paths free of non-conductive deposits, with the lowest possible resistivity, while maintaining shallow path depth. The authors of [18] achieved a lower sheet resistance of 0.04 Ω/sq but with a path depth exceeding 100 µm, whereas our most conductive paths exhibited a sheet resistance of approximately 0.08 Ω/sq with significantly shallower depth in the range of 20–25 µm. It should be noted that the two-stage method does not enhance the electrical conductivity of the path, given that the decomposition process occurs at comparable accumulated fluence values, and—as studies have shown—the second stage of the process only removes the non-conductive layer, enabling the formation of joints. Assuming a sheet resistance of 0.08 Ω/sq and a layer thickness of 12.1 μm, the calculated resistivity of the layer is 0.97 × 10^−6^ Ωm. However, this estimation carries a high degree of uncertainty, which stems from the significant roughness and irregularities in the surface profiles of both the layer and the substrate after the removal of the Al layer (Figure 8). For the Al layer profile (Figure 8a, Profile B), the local deviation from the mean line is estimated at approximately ±4.5 μm, whereas after layer removal, the deviation from the mean line is at the level of ±2.5 μm. Based on these values, the combined thickness uncertainty for a coverage factor of k = 2 is 5.9 μm, representing approximately 50% of the total layer thickness. Consequently, the estimated resistivity value is (0.97 ± 0.49) × 10^−6^ Ωm. While this value is close to published results [10,13,15,16,17,18,21], the high uncertainty in thickness makes a direct comparison difficult.

**Figure 11:**
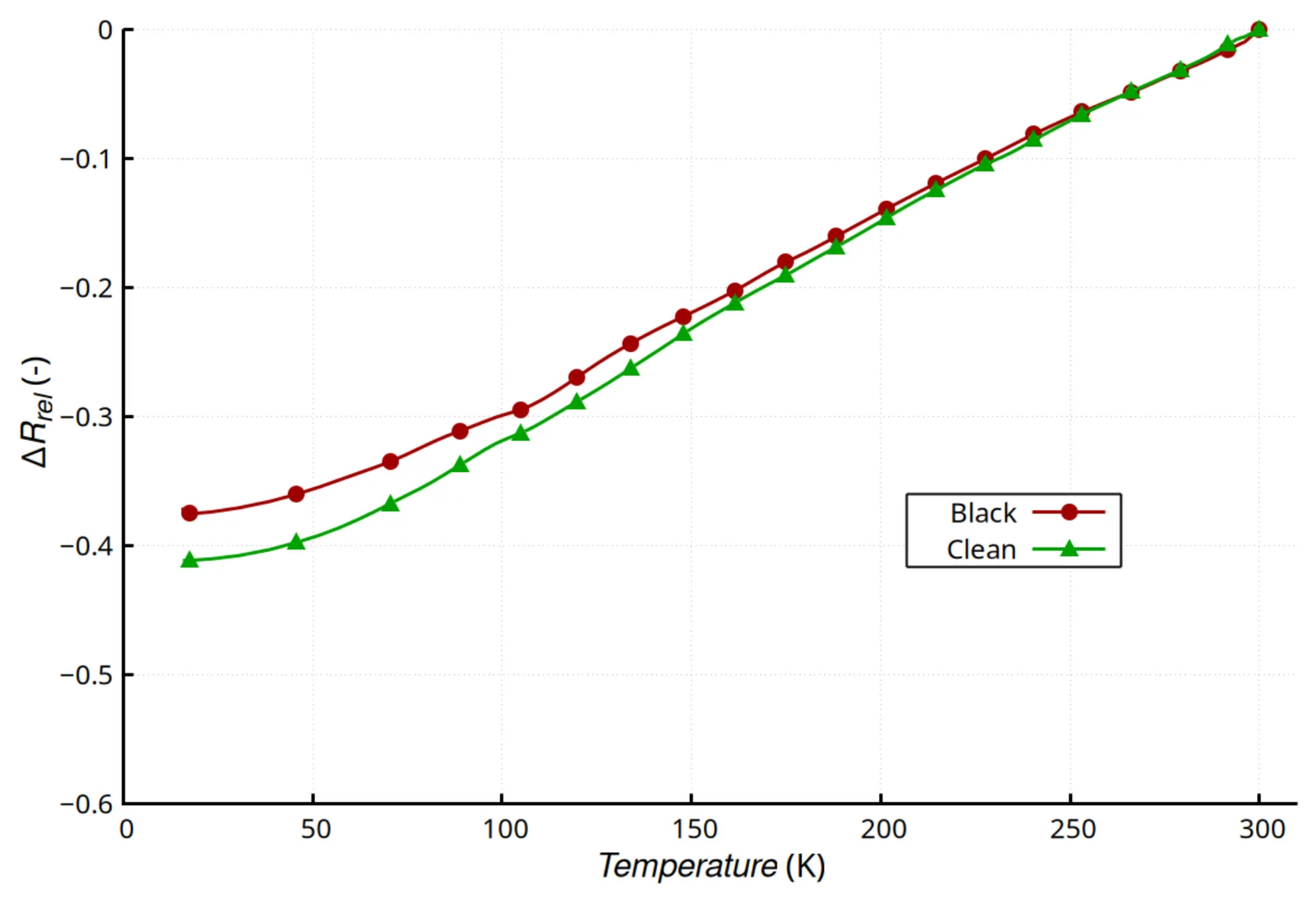
Relative change in the resistance of the Al layer before and after removing the black dielectric layer.

**Figure 12:**
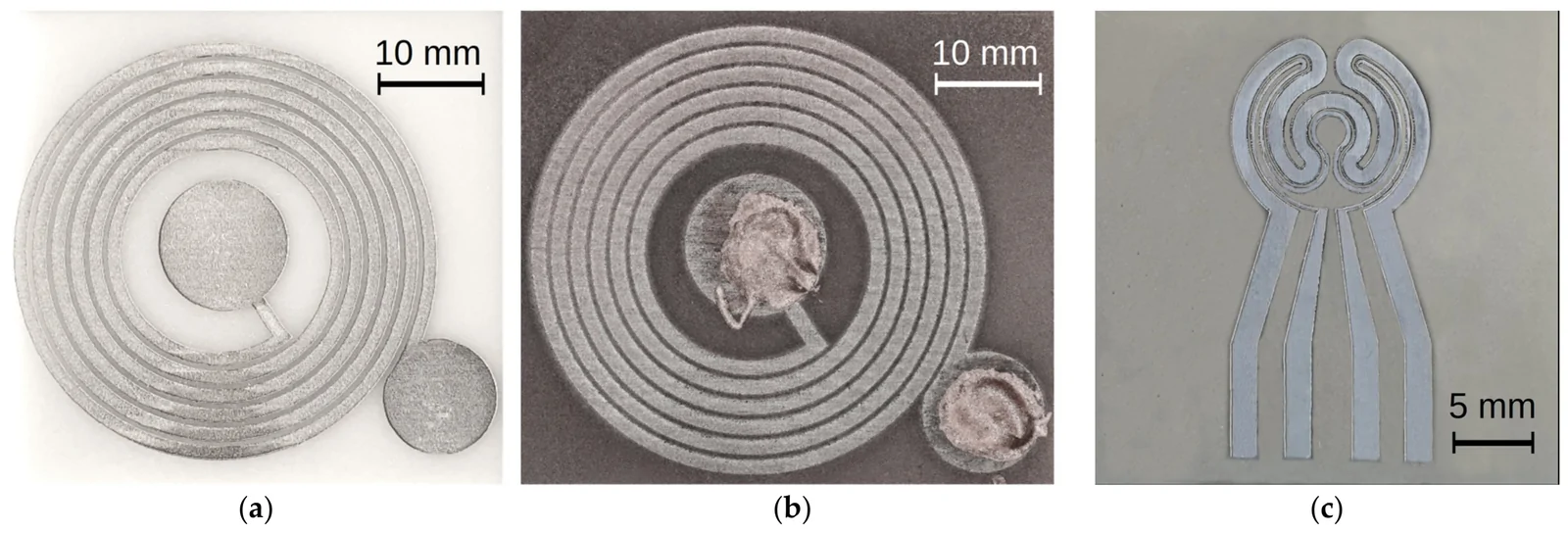
Passive electronic components manufactured by a *two-stage* laser decomposition process of AlN: (**a**) planar coil; (**b**) planar coil with adhesive connection of Ag wire; (**c**) heater structure (15.6 Ω) with a temperature sensor (49.7 Ω).

Figure 11 shows the relative changes in sample resistance before and after removing the black coating layer formed after the *first stage* of the laser treatment process. The measurement of electrical parameters of this layer confirmed the dielectric properties of the black layer, which made it difficult to make good electrical connections to the appropriate conductive Al layer. The *second stage* of the process effectively removed the dielectric layer, but it was necessary to determine the effect of this process on the conductive Al layer. For this purpose, the sample’s resistance was measured over a wide temperature range before and after removing the dielectric layer. It should be noted that the applied process does not affect the type of resistance changes of the sample but only increases its resistance by approximately 3%. This is due to the slight interaction of the laser beam with the conductive Al layer. Changes in layer resistance of the order of 40% were observed compared with the resistance at 300 K, with this relationship being strongly dependent on the laser processing parameters. Optimization and selection of laser processing parameters allowed for the production of layers with parameters similar to typical metallic layers. An example of the application of the developed and optimized technology is the heater sensor structure (Figure 12), which was tested over a wide temperature range. It should be noted that the electrical connections to the tested structure were made using the same electrically conductive paste [28]. No degradation of the connections was observed after repeated heating and cooling.

**Figure 13:**
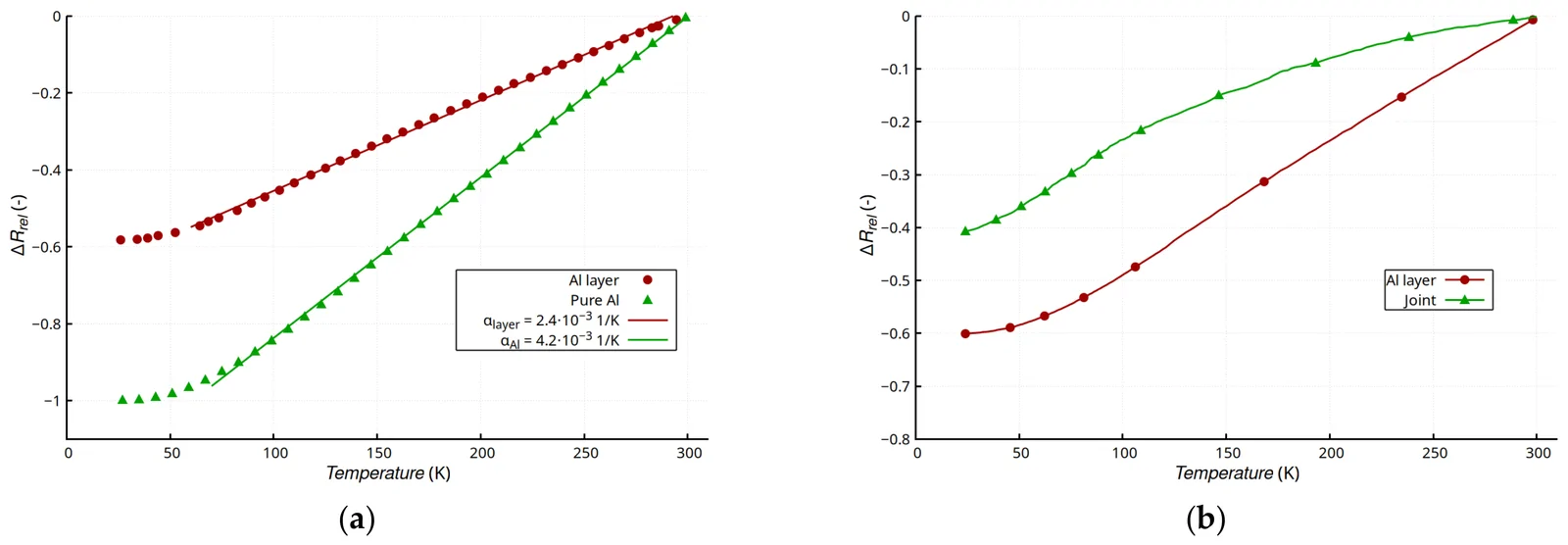
Comparison of relative changes in resistance of: (**a**) Al path and pure aluminum; (**b**) Al layer and adhesive joint.

The geometry of the tested heater temperature sensor system ensures the uniform heating of the ceramic surface in the heater area and, at the same time, the good thermal coupling of the heater temperature sensor. In the first stage of this study, changes in temperature sensor resistance were determined in the range of 20–300 K. Figure 13a shows the results of the relative change in the resistance of the Al path with the relative change in the resistance of pure aluminum. A 60% change in layer resistance was observed, a typical behavior of pure metals.

No discontinuities or unpredictable resistance fluctuations were observed. The temperature coefficient of resistance of Al layers (α_layer_ = 2.4 × 10^−3^ 1/K) is lower compared with pure aluminum (α_Al_ = 4.2 × 10^−3^ 1/K), which results directly from the morphology of the layers. The layer formation process is highly dynamic, resulting in layers that are markedly heterogeneous and contain non-conductive phases, with uneven thickness—factors that significantly affect the temperature coefficient of resistance. The TCR value determined from resistance changes cannot be correlated with temperature-induced changes in the resistivity of the layer material. Both the Al layer and the adhesive joint exhibit a positive TCR value, with the magnitude being lower for the joint (Figure 13b). In the case of the joint, the change in resistance as a function of temperature is non-linear due to the composite nature of the joint material.

**Figure 14:**
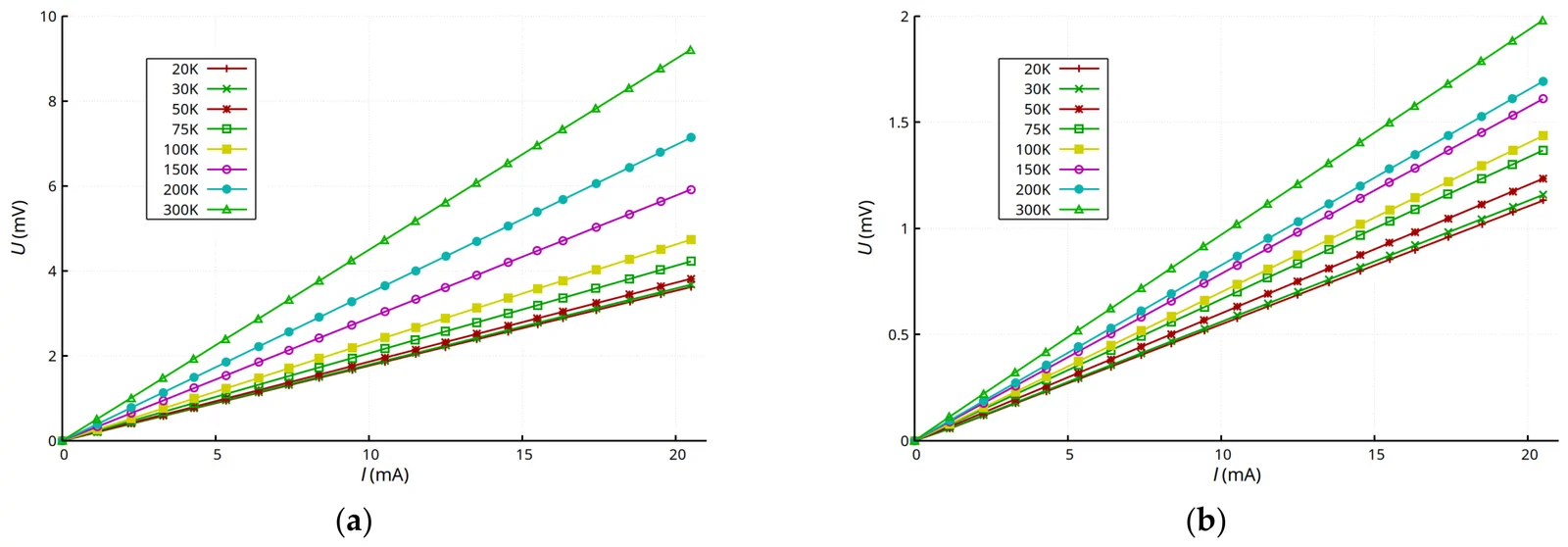
Effect of temperature on U = f(I) characteristics: (**a**) Al layers; (**b**) adhesive joint.

Figure 14 presents the current–voltage (*U* = *f*(*I*)) characteristics of the Al path (a) and the adhesive joint (b). The range of current variations was selected to avoid specimen self-heating. Within the investigated current range (0–20 mA), linear voltage dependence was observed, indicating the ohmic nature of the joint. The resistance of the adhesive joint did not exceed 0.1 Ω, while the resistance of the tested path was approximately 0.45 Ω.

**Figure 15:**
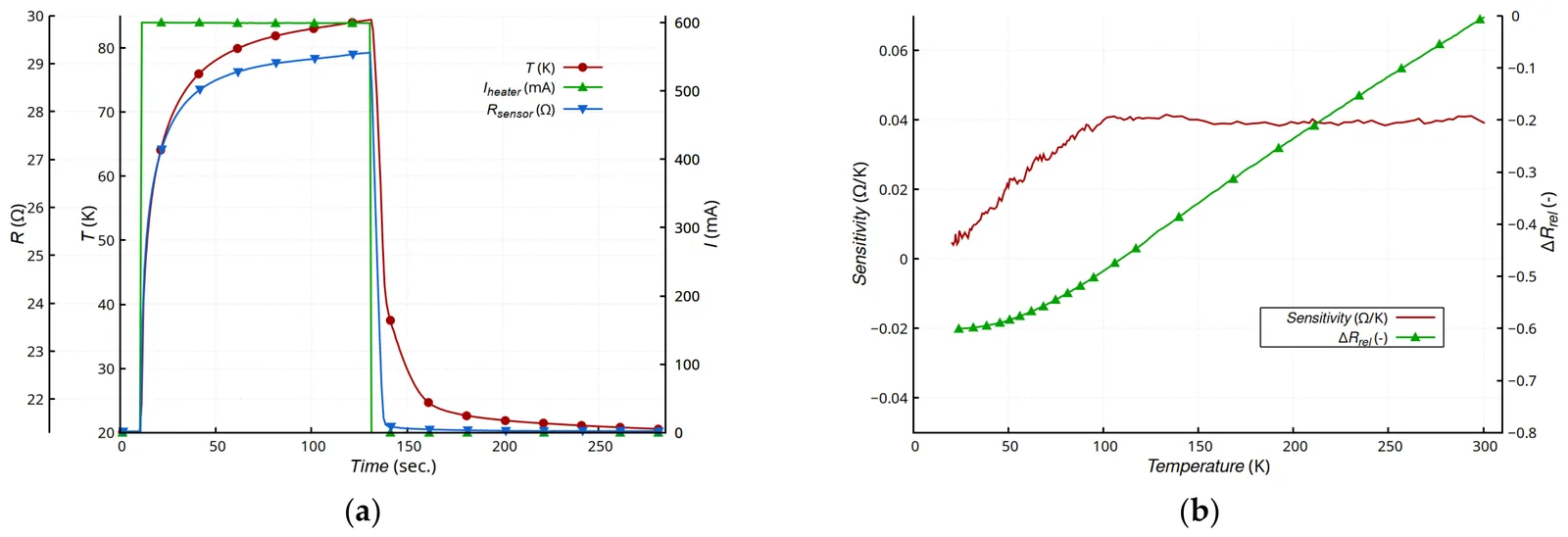
Changes in the electrical properties of Al paths due to the dynamic heating of microstructures (**a**) and effect of temperature on sensitivity and resistance of integrated temperature sensor (**b**).

The next stage of the present research study involved analyzing the system’s dynamics. Current pulses of preset values and duration were applied to the heater. Temperature measurements were performed using a sensor integrated in the structure (Figure 2b and Figure 12c) and an external reference sensor mounted directly on the fabricated structure (DT-670-SD silicon diode). The results presented in Figure 15a indicate good dynamic properties of the heater sensor system. The response of the integrated temperature sensor was similar to that of the reference sensor. Changes in the sensor’s resistance are identical to changes in the temperature indicated by the reference sensor. This is directly related to the very good thermal coupling between the heater and the sensor and the very good thermal properties of the substrate (AlN ceramic).

The heater power in the tested system was 5.6 W at room temperature, while the cooling power of the second stage of the cryocooler was 10 W at 20 K and increased with the rise in temperature. It is important to note that the thermal properties of copper (conductivity and specific heat) undergo significant changes within the temperature range under consideration, which affects heat transfer. The sensitivity of the integrated sensor decreases at low temperatures, a direct consequence of the layer’s *R* = *f*(*T*) characteristic (Figure 15b). Similarly, the heater power decreased, reaching about 3.3 W at 25 K.

It should be emphasized that to verify the thermal stability of the electrical characteristics of the fabricated structures, cryogenic temperature measurements were repeated after 1 year and 2 years. No degradation in path resistance was observed over the two-year evaluation period.

## 4. Conclusions

The two-stage laser decomposition of AlN presented herein enables the precise fabrication of conductive metallic Al paths and passive structures of arbitrary geometries. Maintaining a continuous Argon flow within the processing chamber during both stages is essential to preventing re-oxidation and preserving metallic integrity. The synthesized conductive elements exhibit superior interfacial coupling with the AlN substrate, thereby fully exploiting the exceptional thermal conductivity of the AlN ceramic. Energy-dispersive X-ray spectroscopy (EDS) revealed a high elemental aluminum concentration of 70.6 wt.% within the surface layer—substantially exceeding values previously reported for laser-decomposed AlN. These findings are complemented by X-ray diffraction (XRD) analysis, which confirms the presence of metallic Al; however, due to the X-ray penetration depth probing both the thin Al layer and the bulk substrate, the characteristic Al diffraction peaks remain weak relative to the substrate reflections. In contrast, EDS provides a more representative near-surface quantification owing to its smaller interaction volume (analytical depth of a few micrometers), which matches the thickness of the synthesized layer. Temperature-dependent electrical transport measurements (20–300 K) confirmed the characteristic metallic behavior of the functionalized regions. To evaluate the practical integration of these paths into electrical circuits, the channel depression depth is a critical parameter governing contact formation. The proposed technology allows for the fabrication of embedded Al paths with controllable depths ranging from ~15 µm to ~25 µm at beam overlap factors of *Ox* = *Oy* = 79% and *Ox* = *Oy* = 84%, respectively. Furthermore, successful wire bonding was demonstrated by attaching silver (Ag) leads to the embedded Al paths via conductive adhesive. Ultimately, optimizing the laser decomposition parameters requires a balanced compromise among achieving low electrical resistance, maintaining an acceptable path depression depth, and preserving surface smoothness.

## Figures and Tables

**Table 1:** The variable parameters in the *first and second stages* of the process.

Stage of the Process	Pulse Energy *E_p_* [mJ]	Scanning Velocity *v_s_* [mm/s]	Pulse Overlap *Ox* [%]	Pulse Overlap *Oy* [%]	Accumulated Fluence *F_av_* [J/cm^2^]
First stage of the process	0.170–0.570	80–300	68–92	63–89.5	74–924
Second stage of the process	0.1	300	68	68	43

## Data Availability

The raw data supporting the conclusions of this article will be made available by the authors on request.
